# A Bayesian computational basis for auditory selective attention using head rotation and the interaural time-difference cue

**DOI:** 10.1371/journal.pone.0186104

**Published:** 2017-10-05

**Authors:** Dillon A. Hambrook, Marko Ilievski, Mohamad Mosadeghzad, Matthew Tata

**Affiliations:** Department of Neuroscience, University of Lethbridge, Lethbridge, Alberta, Canada; Australian Research Council Centre of Excellence in Cognition and its Disorders, AUSTRALIA

## Abstract

The process of resolving mixtures of several sounds into their separate individual streams is known as auditory scene analysis and it remains a challenging task for computational systems. It is well-known that animals use binaural differences in arrival time and intensity at the two ears to find the arrival angle of sounds in the azimuthal plane, and this localization function has sometimes been considered sufficient to enable the un-mixing of complex scenes. However, the ability of such systems to resolve distinct sound sources in both space and frequency remains limited. The neural computations for detecting interaural time difference (ITD) have been well studied and have served as the inspiration for computational auditory scene analysis systems, however a crucial limitation of ITD models is that they produce ambiguous or “phantom” images in the scene. This has been thought to limit their usefulness at frequencies above about 1khz in humans. We present a simple Bayesian model and an implementation on a robot that uses ITD information recursively. The model makes use of head rotations to show that ITD information is sufficient to unambiguously resolve sound sources in both space and frequency. Contrary to commonly held assumptions about sound localization, we show that the ITD cue used with high-frequency sound can provide accurate and unambiguous localization and resolution of competing sounds. Our findings suggest that an “active hearing” approach could be useful in robotic systems that operate in natural, noisy settings. We also suggest that neurophysiological models of sound localization in animals could benefit from revision to include the influence of top-down memory and sensorimotor integration across head rotations.

## Introduction

In natural settings, sounds emanating from different sources mix and interfere before they reach the ears of a listener. The process of resolving individual sound sources is known as auditory scene analysis and constitutes a set of unsolved problems in psychology, computer science, and neuroscience. Decades of psychological study have revealed that auditory scene analysis occurs in three interacting dimensions: frequency, time, and space. In the frequency domain, sounds are separated monaurally at the cochlea, whereas the spatial representation of distinct sound sources emerges later in the auditory system primarily through comparisons between signals from the two ears. Early decomposition of the spectral and spatial features of the auditory scene is then resolved by a poorly understood mechanism that binds related features together to form perceptual streams [1]—mental representations of sound sources distinct from each other and from the background. Resolution of individual acoustic streams is the result of fusion of sound components that fit together based on heuristics that combine information along all three dimensions. While evolution has arrived at solutions for solving the problem presented by complex acoustic scenes, it remains an obstacle in computer science and robotics, one that must be overcome before the goal of systems that can interact naturally with the world can be achieved.

**Fig 1:**
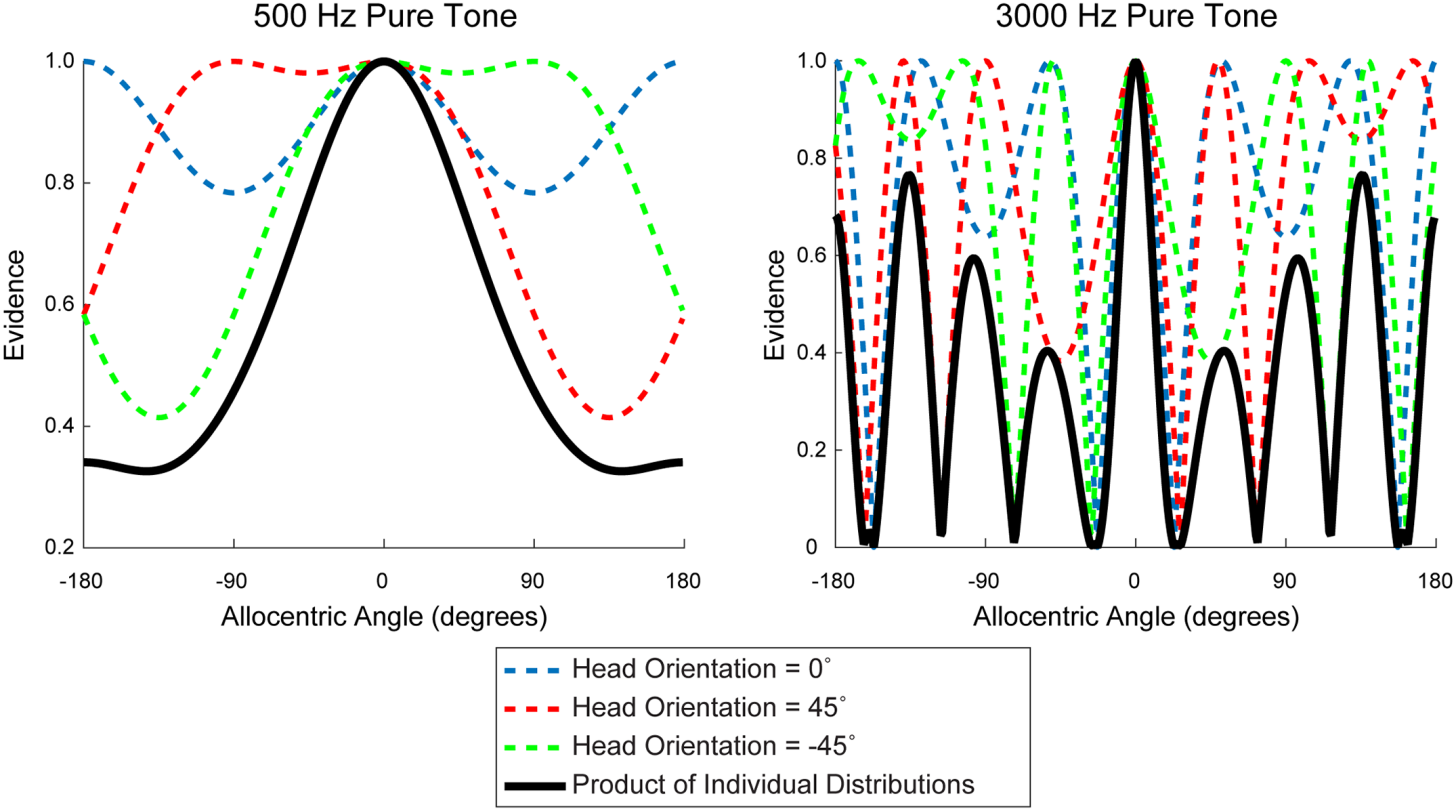
Modelled beamformer response to pure tones at varying azimuth angles in egocentric space. Response to a modelled sound source located at 0° in allocentric space from a set of narrow-band beamformers oriented at various angles in egocentric space obtained from Eq 1. At low frequencies, peak activation occurs over broad arcs; multiplying evidence distributions across successive rotations results in a reduced localization uncertainty. At high frequencies multiple peaks are eliminated by multiplying across rotations effectively resolving ambiguity.

Two important cues to sound location, particularly the azimuthal angle of arrival, are inter-aural time difference (ITD) and inter-aural level difference (ILD). A substantial body of literature has found classes of neurons that represent spatial information by encoding one or both of these cues in both birds [2,3] and mammals [4–7]. This literature confirms the canonical duplex theory of sound localisation: Ensembles of neurons sensitive to high-frequency sound components compute location using ILD, while low-frequency sensitive neurons rely on ITD. This mechanism of localisation is memoryless and egocentric—units decode spatial information from immediately available bottom-up signals in head-centred coordinates [8]. An important and well-known limitation to such systems is that they result in ambiguity: First, both ILD and ITD cues are ambiguous with respect to front/back localization (the ILD and ITD cues are identical for sound sources at opposite azimuths across the interaural axis). Second, for high-frequency sounds such that the interaural distance is greater than one-half the wavelength of the sound, ITD yields additional “phantom” images due to spatial aliasing of phase-delayed signals (See Fig 1). For high frequencies and transiently stationary signals, a number of tightly-spaced side lobes may exist because multiple interaural time delays can equivalently account for the observed phase lag. For a given true arrival angle of sound, the relative signal strength appearing to arrive from a source at these “angles of confusion” is given by Eq 1:

$$A\left(\psi ,\theta \right)=\frac{\sqrt{2+2\text{cos}\left(2\pi fd\left(\frac{\text{sin}\left(\psi \right)-\text{sin}\left(\theta \right)}{c}\right)\right)}}{2}$$ (1)

Where *f* is the frequency of the sound, *c* is the speed of sound, *d* is the distance between microphones, *ψ* is the arrival angle of the sound measured from the frontal midline, and *θ* is the steering angle for a particular beam. We show here that these ambiguities are well-resolved in an active system with memory.

Artificial hearing systems such as in robotics face the same computational problems as biological systems with respect to spatial ambiguity, thus understanding biological solutions helps to inform artificial solutions. A large part of the prior research in the neurobiology of auditory localization has assumed that the head does not rotate. In this case the egocentric (i.e. self-referential) and allocentric (i.e. externally referenced) spatial representations of the world stay aligned. However, several studies have suggested that active rotation of the head probably adds substantial information about the auditory scene, particularly with respect to the ambiguities associated with binaural hearing. Hans Wallach [9] demonstrated that head rotations could be useful in resolving not only front-back ambiguity, but the entire three-dimensional cone of confusion. In another study [10] he showed that vestibular cues during head rotation are sufficient to improve sound source resolution, but that visual cues also contribute. Burger [11] showed that restricting head movements impairs listeners ability to discriminate front from back field sources although not to chance levels. Interestingly, listeners responded near chance when one ear was masked, suggesting that binaural cues, rather than monaural spectral cues due to the pinnae, are necessary to make use of head rotations. Thurlow et al. [12] observed and characterized listeners’ natural head movements while localizing sounds and found that listeners make substantial rotations about the vertical axis as well as reversals of rotation. Thurlow & Runge [13] induced head movements in a localization task and found that rotations reduced azimuthal errors. Perret & Noble [14], Wightman & Kistler [15], and Hill et al. [16] likewise showed strong evidence that active head movements aid sound source localization, particularly by resolving front/back ambiguity. Brimjoin et al. [17] reproduced Wallach’s original 1940 demonstration that head rotation coupled with sound source movement can influence perceived location such that an illusory stationary source appears in the front/back field opposite the true source; this is the so-called “Wallach Effect” or “Wallach Illusion”. Finally, distortion of the vestibular signal was shown to distort sound localization [18] suggesting that integration of egocentric auditory cues into an allocentric frame of reference established by other senses is important for effective sound localization. This prior literature strongly supports the notion that humans use an “active hearing” approach to localizing sound. We propose here a model of how the auditory system uses head position to integrate the instantaneous egocentric evidence provided by binaural cues with a prior allocentric representation of probable sound sources. Our approach is motivated broadly by the notion that the brain maintains and updates a generative model of the sensory scene in a Bayesian fashion [19–22].

The advantages provided by active hearing have been especially important in recent literature on computational auditory scene analysis, particularly in the field of robotics. The goal of this work has been to improve the spatial resolution of the auditory scene analysis by rotating or translating the microphones (ears) of a robot over time. A number of successful approaches have been described using both multi-microphone arrays [23–27] and also binaural systems [28–33]. What each of these approaches has in common is an algorithm by which instantaneous evidence and prior knowledge are co-registered over successive movements of the microphones (for example by Recursive Bayesian, Kallman or Particle Filtering approaches).

Prior work on spatial decomposition of the auditory scene has emphasized localization—the ability of the model or system to find one or more sources in space. However attentional selection of a sound source requires the further step of resolving its frequency components from distractors and the background. It has generally not been considered whether ITD (or ILD) models can also effectively decompose the scene in the frequency domain. However, there is reason to suspect that a system comprised of ITD sensitive units that have some frequency selectivity might separate sounds in both the spatial and frequency domains. ITD sensitive cells are known to exhibit some degree of frequency tuning in both birds [3] and mammals [7]. In the computational scene analysis literature, Roman et al. [34] showed a binaural model system based on ITD and ILD that found spatially distinct sound sources in both frequency and space. Here we demonstrate that a canonical binaural approach to localization based solely on ITD (employing banks of frequency-tuned delay-and-sum beamformers) not only successfully localizes single sound sources but also resolves frequency components from mixtures. The resulting spectral-spatial map provides the basis for auditory selective attention, by providing not only the location of the individual sound sources but also information about their frequency content. The model has two basic requirements: first, the head must be allowed to rotate; second, a prior memory (on the time scale of a few seconds) for spectral and spatial information must be integrated with bottom-up sensory evidence. Notably, resolution of individual acoustic streams is not limited to low-frequencies, as might be expected from a method employing only two sensors and exclusively ITD cues, nor is it limited by front/back confusion. If allowed to rotate, a binaural beamformer system builds very accurate and unambiguous maps of the entire azimuthal auditory scene over successive rotations. Here we demonstrate a real-time implementation on a simple auditory robot.

## Methods

Our goal was to arrive at an allocentric map (i.e. in world coordinates) of the auditory scene in which frequency and space are represented independently and activity in the map represents a long-running estimation of the likelihood that a sound source occupies particular spectral and spatial locations. However, the acoustic input to the ears (or pair of microphones) is egocentric with respect to the head and rotates through allocentric space as the head is turned. We used a Bayesian approach that merges bottom-up instantaneous egocentric information with an allocentric memory of the auditory scene built over successive rotations of the head.

### Audio capture and the physical space

We explored the importance of head rotation using a custom built simple robot with a pair of microphones that could be rotated through azimuthal angles. Cardioid microphones, (2 Apex 150 Miniature Condenser Microphones) were fixed to a horizontal bar with 0.145 m spacing. Azimuthal angle of the microphone pair was controlled with an Arduino Uno microcontroller and an Adafruit Motor Shield V2 driving a stepper motor in “microstep” mode. Control of the allocentric angle of the microphone pair was integrated with the scene analysis computation via the MATLAB Arduino Hardware Support Add-On.

Artificial stimuli (noise, tones and tone complexes) were generated with Praat [35]. Speech stimuli were drawn from the Pacific Northwest/Northern Cities corpus [36]. Stimuli were presented on Mackie HR624 studio monitors in a reverberation attenuated space with a constant background noise floor of approximately 60dBA. The broadband RMS amplitude of stimuli presented together was equalized prior to presentation. The distance from the centre of the microphone array to the front of the speakers was 1.1 m. Presentation of stimuli was controlled by the PsychPortAudio module of Psychophysics Toolbox for MATLAB [37]. Playback sampling rate was set to 44100 Hz for all stimuli.

Each experiment consisted of 9 rotations as the robot scanned linearly from 0° to 180° in 20° steps. Pilot experiments that used smaller rotations, and that scanned smaller or different arcs yielded qualitatively similar results.

Left and right audio signals were captured with microphones oriented vertically (orthogonal to the plane through which the array could rotate) and connected to a FocusRite Scarlette USB audio interface. Audio was captured on a MacbookPro computer with a recording sample rate of 48000 Hz and bit-depth of 24 bits. Custom audio capture code written using Apple’s AudioQueue Services took audio buffers of 4096 samples from the left and right channels, added time stamps, cast the channel data into double precision and dumped the interleaved data into shared memory for MATLAB’s memmap() function to pick up.

### Spectral and spatial decomposition of the short-time scene

In the present study we sought only to demonstrate the potential for high-resolution auditory scene analysis by a system using ITD as a cue. Although there exist several well-developed models of the early auditory system (see, for example, [38]), we did not set out to validate any particular model of the early auditory system. Instead we chose a generic approach with acknowledged stages of spectral followed by spatial decomposition.

We approximated the filter bank function of the basilar membrane with a gammatone filter bank [39] with 50 linear spaced centre frequencies between 100 Hz and 5000 Hz. Equivalent rectangular bandwidth (ERB) scale spaced centre frequencies over the same range yielded qualitatively similar results but were less intuitive to visualize in figures. Each time frame of 4096 audio samples was filtered in real time using a custom MEX MATLAB function, resulting in a 2 (channels) x 50 (bands) x 4096 (samples) data structure.

**Fig 2:**
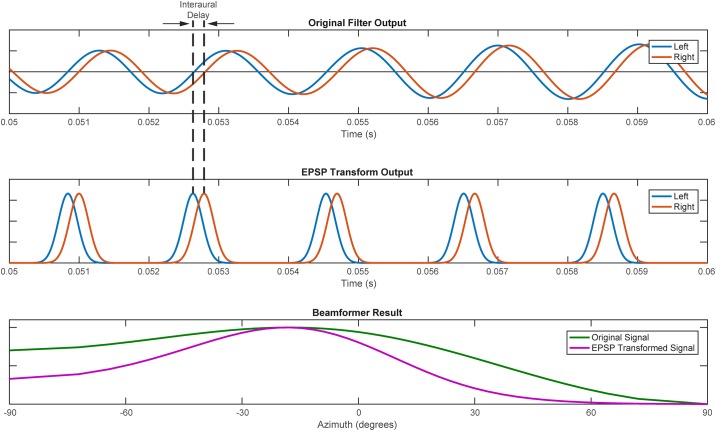
Illustration of the EPSP transform and its effect on beamformer results. Example of the transformation of a sound signal filtered at 500 Hz (top panel) into an excitatory postsynaptic potential (EPSP) signal (middle panel). The EPSP transform results in a relatively narrower beamformer-array output that is stable with respect to frequency of the filter pass-band (bottom panel).

We sought only to demonstrate an approach using ITD as a cue to sound location. We thus approximated the canonical Jeffress circuit of delay lines and coincidence detectors [2] as banks of frequency-tuned delay-and-sum beamformers [40]. Coincidence detection neurons in both birds and mammals rely on precisely timed excitatory postsynaptic potentials (EPSPs) arriving from both ears that are phase-locked relative to the incoming sound waveform. To replicate this EPSP output the filtered audio signal was transformed by creating an analogous signal that consisted of all zeros except at samples that correspond to positive zero-crossings in the audio signal, where the value was set to one; this binary signal was then convolved with a 1 ms wide Gaussian window, modelled after EPSPs recorded from the medial superior olive of Mongolian gerbils, as illustrated in Fig 2 [41].

The delay-and-sum beamformer stage arrives at a set of “monaural” signals for each frequency band by steering a beam into each available angle. Conceptually, each beam angle thus models an individual coincidence detector in the canonical Jeffress circuit. The number of beams that can be computed is twice the number of beams per hemifield, plus a centre beam. The number of beams in a hemifield is the product of the distance between microphones and the sampling rate, divided by the speed of sound. The angles of arrival for each beam are therefore given by Eq 2:

$$\theta =\text{Re}\left({\text{sin}}^{-1}\left(\frac{cT_{b}}{d}\right)\right)$$ (2)

where *T*_*b*_ is the characteristic time delay for a given beam and *d* is the distance between microphones.

Each beam is then computed by time shifting the left and right channels, *n*, relative to each other using the time delay, in samples, appropriate for the angle of each beam, given by the product of *T*_*b*_ and the sampling rate *f*_*s*_, and then summing the channels, *y*_*b*,*n*_. This delay-and-sum approach temporally aligns a signal present in both the left and right ears by accounting for the time difference of arrival due to different path lengths between the source and each ear, and is computationally analogous to the system of delay lines and coincidence detectors [2,3]. The resulting time-series beam, *Z*_*b*_, is computed by Eq 3:

$$\left\{ \begin{array}{l} y_{b,n}\left(k\right)=y_{n}\left(k+f_{s}T_{b}\right)\\ Z_{b}\left(k\right)=\frac{1}{N}\sum_{n=0}^{N}y_{b,n}\left(k\right) \end{array}\right.$$ (3)

The beamformer stage collapses left and right channels but yields an output matrix of 50 (bands) x 41 (beams) x 4096 (samples). This bands x beams x samples matrix was then collapsed by estimating the energy within each beam by computing the RMS of the signal over the *N*_*k*_ = 4096 time samples (Eq 4) creating an egocentric image, *S*, of band *f* and beam *b*:

$$S_{f,b}=\sqrt{\frac{\sum_{k}Z_{f,b}{\left(k\right)}^{2}}{N_{k}}}$$ (4)

The resulting 2-dimensional “map” with frequency on one axis and spatial angle on the other captures a short-time (~83 ms) spectral and spatial representation of the auditory scene in egocentric coordinates. The energy within each location in this map constitutes evidence that a sound source occupies a particular frequency and spatial angle. This egocentric map will contain ambiguous information about the location of a sound source however the ambiguity can be resolved by comparing the map to others obtained at different head orientations.

### Computation of a probabilistic map in allocentric space using recursive Bayesian algorithm

To resolve the ambiguities associated with ITD it is sufficient to notice that a beam pointed at the true arrival angle of a sound source in the allocentric world always has peak sensitivity to that sound regardless of the frequency of the sound or the orientation of the head. By contrast, the set of ambiguous lobes may “point to” different locations in the allocentric world as the head is rotated. A system that has memory can resolve this ambiguity by rotating the head and co-registering the different egocentric input into an allocentric map.

We modelled such a system using a simple recursive Bayesian approach. Memory for the positions of sound in the spectral-spatial angle map constituted a distribution of prior probabilities, while incoming short-time maps (containing ambiguity) constituted new evidence. With each iteration the algorithm multiplies the new evidence with the prior memory and arrives at a new posterior distribution of probabilities. This posterior map is then used recursively as the prior map for the next iteration.

Front/Back information is redundant in the egocentric space around a two-microphone (or two ear) array. Thus, each incoming egocentric spectral-spatial map was reflected into the space behind the axis of the microphone array. Since the widths of beams in a delay-and-sum beamformer are not uniform around the circle (peripheral beams are wider than midline beams) we used spline interpolation to convert the incoming maps into maps with 360 evenly spaced angles around the circle. The egocentric map, *PDF*^*ego*^, was normalized so that the beams within any single frequency band summed to one (Eq 5).

$$PDF^{ego}\left(f,b\right)=\frac{S_{f.b}}{\sum_{b=0}^{b=B}S_{f,b}}$$ (5)

Finally, the egocentric map obtained in Eq 5 was rotated by the negative of the head orientation to align each incoming map with allocentric space, *PDF*^*allo*^. For each incoming map we updated the allocentric map of prior probabilities by multiplying the previous prior map with the incoming evidence.

$$PDF^{allo}=\prod_{r=0}^{r=R}PDF_{r}^{allo}$$ (6)

If there was not an existing allocentric map, a map was initialized as an array of ones. This resulted in a 360-degree allocentric map representing the likelihood that any particular spectral-spatial location contained a component of a sound source. Importantly, this map updated (and became less ambiguous) over time with successive rotations of the microphones.

### Quantifying system performance

**Fig 3:**
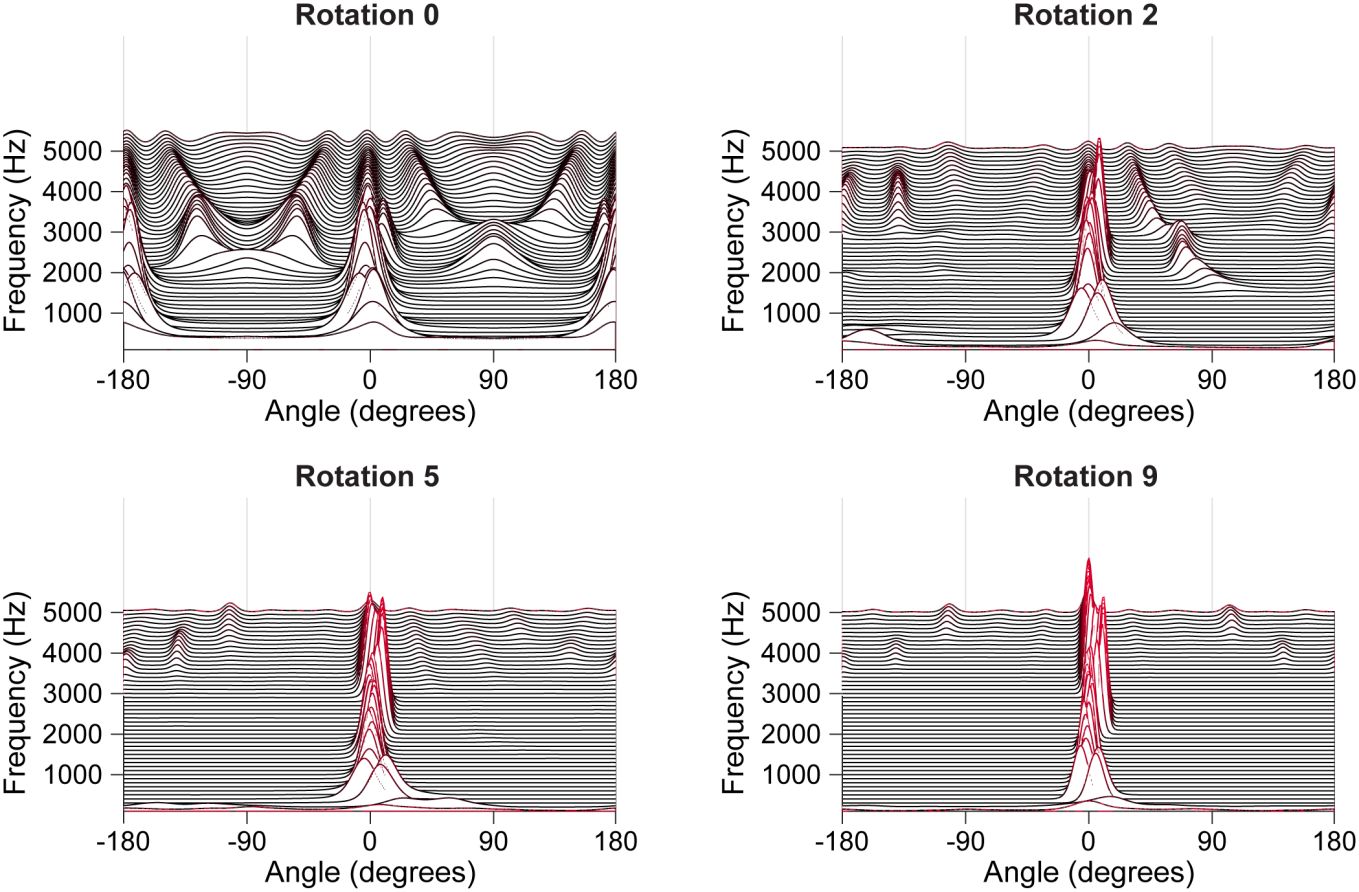
Localization becomes unambiguous over several rotations. Response of a set of narrow-band beamformers to broadband noise at 0° and a 2400 Hz tone at 11° in allocentric space product integrated over several rotations.

**Fig 4:**
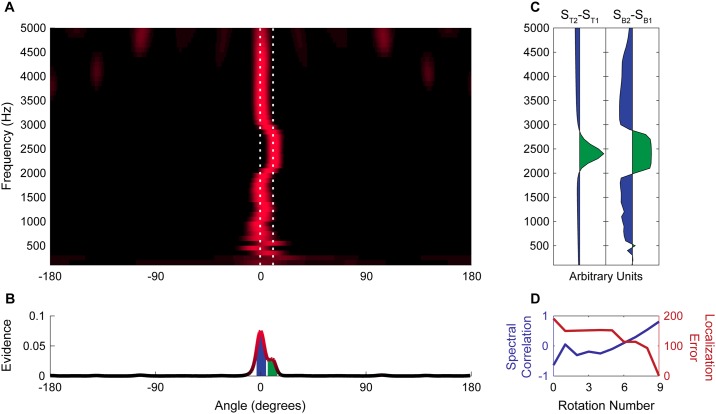
Resolution of broadband noise and a pure tone. (A) Spectral-spatial map of acoustic scene containing broadband noise at 0° and a 2400 Hz tone at 11° after 9 rotations. White dashed lines indicate the true location of sources. (B) Spatial map of the acoustic scene shows two distinct peaks corresponding to the noise and tone, respectively. Filled regions under peaks indicate the extent of the beam patterns used to estimate spectra in panel C. (C) Plots of the difference between the average spectrum of the tone (S_T2_) and broadband noise (S_T1_) (Left) and the difference between the activity in the spectral-spatial map at the peaks shown in panel B near 11° (S_B2_) and 0° (S_B1_). (D) Correlation between difference in the spectra of the target acoustic streams and the difference between the spectra of the two most prominent spatial peaks (blue) and the total localization error between targets and peaks (red) over successive rotations.

To quantify the ability of the system to resolve complex sounds originating at different locations we used two measures: localization error and spectral correlation. For illustrative purposes, we refer to Figs 3 and 4, in which the system resolves a pure tone and broadband noise. Allocentric spectral-spatial maps change across rotations (Fig 3). Localization improvement typically reaches an asymptote after nine rotations and this map is presented as representative of system performance (Fig 4A). Localization error (Fig 4D, red) was determined by identifying the two most prominent peaks in the allocentric map after collapsing across frequency (Fig 4B), calculating the distance between the known location of a sound source and the location of the nearest peak, and adding together the absolute value of that distance for each source in the scene. Spectral correlation (Fig 4D, blue) was determined by calculating the correlation between the difference in average spectral power between the two original target waveforms in the acoustic scene (Fig 4C, left panel) and the difference in unmixed ‘spectral power’ within a range of beams around the two most prominent peaks in the collapsed spectral map (Fig 4C, right). It is important to note that while we refer to the ‘spectral power’ within beams, because the map consists of normalized probabilities rather than real power values it is in fact only analogous to the spectra. These measures reflect the accuracy of the system at localizing distinct sound sources and at resolving their complex spectral content.

## Results

### Resolving pure tones in noise

**Fig 5:**
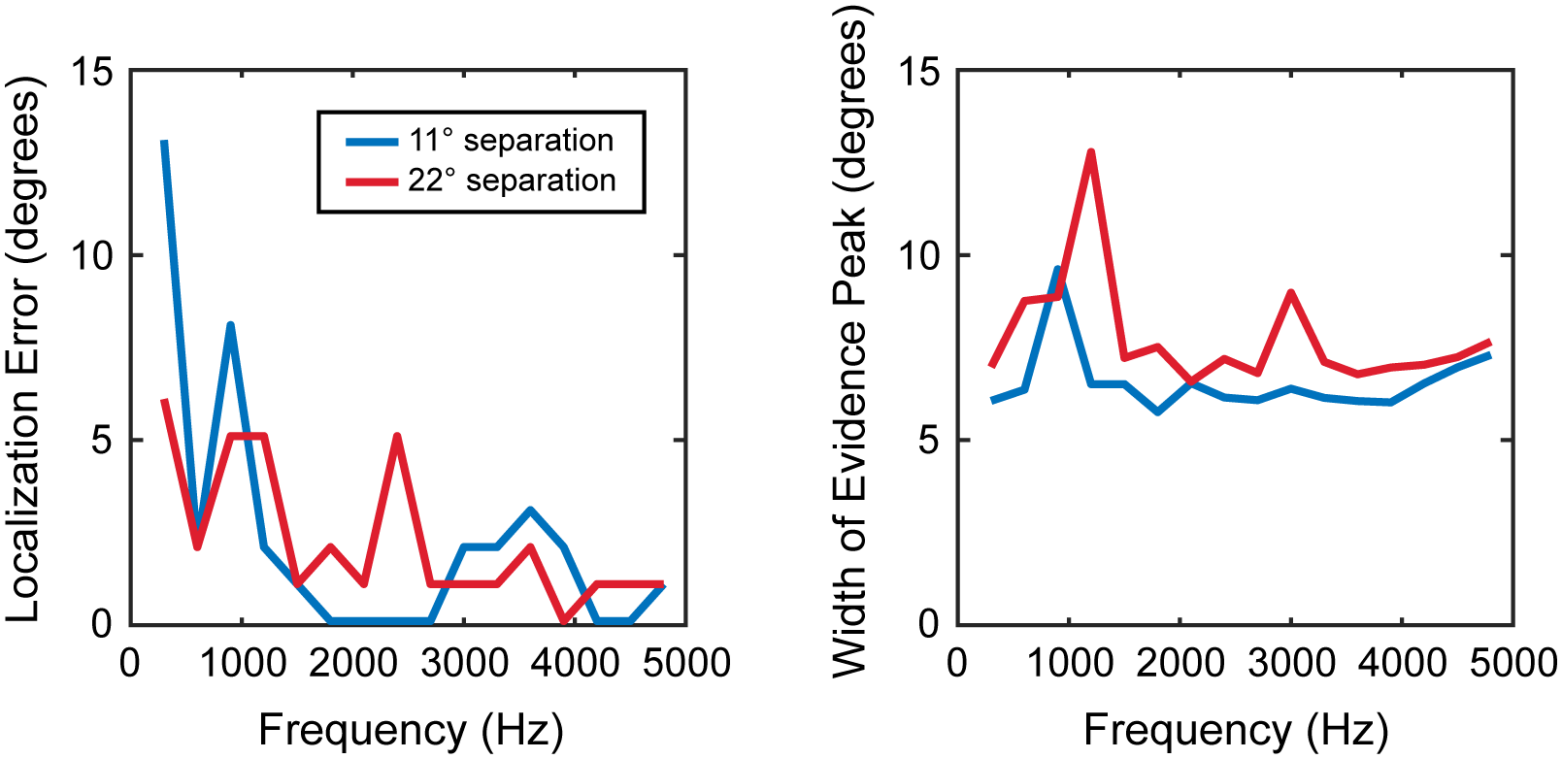
Accuracy and precision of localization is better at high frequencies. Spectral-spatial maps for acoustic scenes with broadband noise at 0° and tones at 11° or 22° were obtained over 9 rotations. Localization error, illustrating the limits of system’s accuracy, and the width of the spatial peak, illustrating the limits of the system’s precision, after 9 rotations are plotted against the frequency of the target tone.

To characterize the performance of the system to resolve sound components at different frequencies we performed resolution runs lasting for 9 rotations in acoustic scenes consisting of broadband noise at 0° and a pure tone at 11° or 22°. Runs were performed with pure tones at frequencies between 300 and 4800 Hz. Fig 3 illustrates how the allocentric spectral-spatial map evolves over rotations for a typical run for a pure tone at 2400 Hz. Prior to any rotations the low-frequency components of the broadband noise are localized accurately, although with considerable front-back confusion. At high frequencies there are numerous spurious peaks at locations without real sound sources. Over successive rotations of the microphone pair the modelled scene converges towards separately localizing both the noise and tone. Fig 4A shows the final spectral-spatial map after 9 rotations for the acoustic scene with broadband noise and a 2400 Hz tone. The deflection away from 0° around 2400 Hz represents the resolution of the tone. Collapsing across frequency bands (Fig 4B) shows that two distinct peaks occur near 0° and 11°. If we compare the difference between the frequency content of the noise and tone with the beamformer activation at different frequency bands along the 0° and 11° beams it becomes clear that the system has resolved the frequency content of the two targets because the difference between spectra of the estimated targets (Fig 4C, right) closely matches the difference between spectra of the original targets (Fig 4C, left). Fig 4D shows how resolution is improved by rotation: the spectral correlation between the target sound sources and the resolved sound sources increases with rotations and the localization error, that is the difference between the proposed and actual sound source locations, is reduced. These results are typical for acoustic scenes with high frequency targets (Fig 5). The system is slightly less accurate at resolving very low-frequency (<600 Hz) targets; low frequency tones remain unresolved from the broadband noise at 11° separation, however they can be reliably resolved when the physical separation is increased to 22°. Tones in noise can be reliably localized to within 5° of their true location when separated from the noise by more than the typical peak width.

### Resolving tone complexes

**Fig 6:**
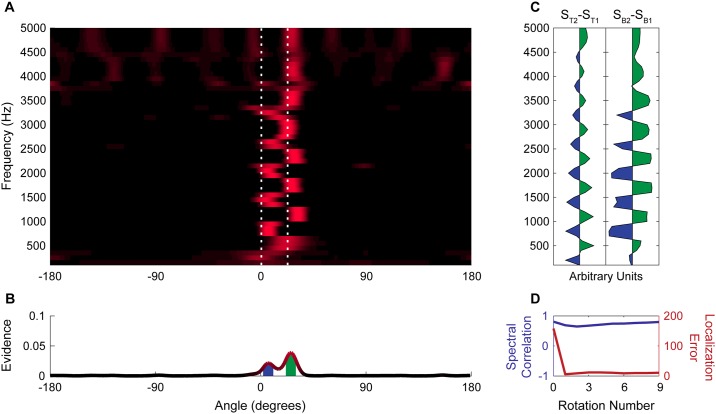
Resolution of two complex tones spatially separated by 22°. (A) Spectral-spatial map of acoustic scene containing tone complexes at 0° and 22° after 9 rotations. White dashed lines indicate the true location of sources. (B) Spatial map of the acoustic scene shows two distinct peaks corresponding to the two tone complexes. Filled regions under peaks indicate the extent of the beam patterns used to estimate spectra in panel C. (C) Plots of the difference between the average spectrum of the two tone complexes (Left) and the difference between the activity in the spectral-spatial map at the peaks shown in panel B near 22° (S_B2_) and 0° (S_B1_). (D) Correlation between difference in the spectra of the target acoustic streams and the difference between the spectra of the two most prominent spatial peaks (blue) and the total localization error between targets and peaks (red) over successive rotations.

**Fig 7:**
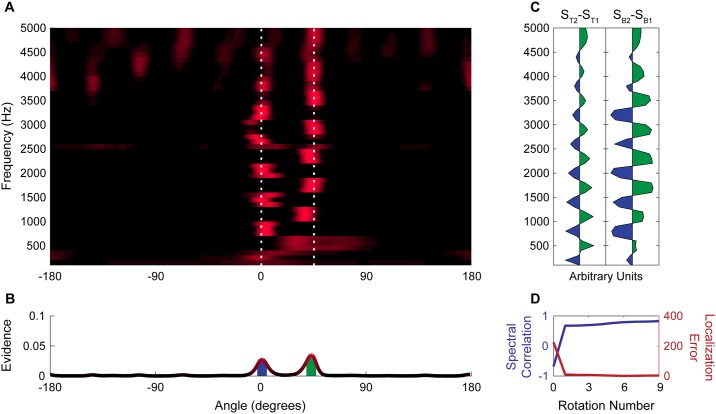
Resolution of two complex tones separated by 45°. (A) Spectral-spatial map of acoustic scene containing tone complexes at 0° and 45° after 9 rotations. White dashed lines indicate the true location of sources. (B) Spatial map of the acoustic scene shows two distinct peaks corresponding to the two tone complexes. Filled regions under peaks indicate the extent of the beam patterns used to estimate spectra in panel C. (C) Plots of the difference between the average spectrum of the two tone complexes (Left) and the difference between the activity in the spectral-spatial map at the peaks shown in panel B near 45° (S_B2_) and 0° (S_B1_). (D) Correlation between difference in the spectra of the target acoustic streams and the difference between the spectra of the two most prominent spatial peaks (blue) and the total localization error between targets and peaks (red) over successive rotations.

To characterize the system’s ability to resolve the spectral-spatial characteristics of more complex sounds, two tone complexes were synthesized that consisted of several pure tones separated by 600 Hz steps starting with, as their lowest frequency components, 200 Hz and 500 Hz respectively and having their highest frequency components at 4400 and 4700 Hz. Resolution runs lasting for 9 rotations were performed for acoustic scenes in which the complexes were separated by 22° (Fig 6) and 45° (Fig 7). In both scenes the system performs well, localizing the tone-complexes to within a few degrees in allocentric space and resolving the comb-like spectral structures of the individual tone complexes. Comparing the results of the two experiments we see that as the spatial separation of sounds in the scene increases, the ability to precisely localize the sound sources and resolve their spectral content improves.

### Resolving speech in multi-talker scenes

**Fig 8:**
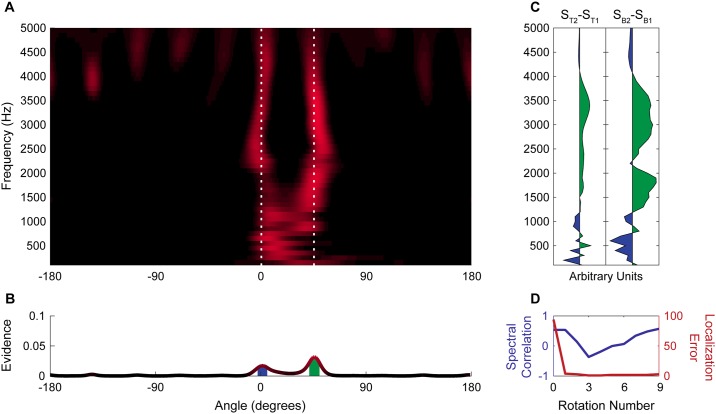
Resolution of two talkers separated by 45°. (A) Spectral-spatial map of acoustic scene containing a female and male speaker at 0° and 45° after 9 rotations. White dashed lines indicate the true location of sources. (B) Spatial map of the acoustic scene shows two distinct peaks corresponding to the two talkers. Filled regions under peaks indicate the extent of the beam patterns used to estimate spectra in panel C. (C) Plots of the difference between the average spectrum of the two talkers (Left) and the difference between the activity in the spectral-spatial map at the peaks shown in panel B near 45° (S_B2_) and 0° (S_B1_). (D) Correlation between difference in the spectra of the target acoustic streams and the difference between the spectra of the two most prominent spatial peaks (blue) and the total localization error between targets and peaks (red) over successive rotations.

To characterize the system’s ability to resolve individual talkers in a complex acoustic scene we performed a resolution run in a scene containing a female and male speaker separated by 45° (Fig 8). The system was able to accurately localize the two speakers and successfully resolved the high-frequency components of the two speakers’ voices. While the spectral differences that are identified by the system may not be well-suited for reconstructing cleanly separated speech, no attempt has been made to optimize the system to achieve that goal; this only represents a proof-of-concept that may be expanded to improve speech recognition in complex acoustic scenes.

## Discussion

The computational model described in this study provides two insights into auditory scene analysis in both biological and machine systems: First, we show that the ITD cue can, under certain circumstances, provide unambiguous sound source localization at arbitrarily high frequencies. Second, we show that the canonical Jeffress circuit might provide a computational basis for both spatially and spectrally selective attention by unmixing complex auditory scenes into spatial and spectral components.

### ITD and resolving spatial ambiguity in biological systems

Across many species, sound source localization is known to involve the comparison of sounds at both ears for arrival time (ITD) and sound level (ILD) differences. The duplex theory of localization holds that ITD is used predominately at low-frequencies whereas ILD is used at relatively higher frequencies. This theory aligns well with physiological evidence and is well accepted. It is sometimes suggested however that binaural systems, both biological and artificial, benefit from deemphasizing ITD in favor of ILD at higher frequencies because of the inherent ambiguities associated with ITD at higher frequencies and the fact that biological systems fail to represent fine structure at arbitrarily high frequencies [7,42,43]. In this study we showed that a generic binaural system that is solely sensitive to ITD can successfully localize and resolve individual sources in a complex auditory scene even at frequencies well beyond the presumed limit of usefulness for the ITD cue. In fact, high frequency information, particularly at frequencies that provide instantaneously ambiguous localization cues in a static system, allows for very accurate localization when head rotation is allowed. This result does not in any way call into question the duplex theory. Our results suggest a more nuanced understanding: in general, high-frequency signals do yield transiently ambiguous localization, however a system that employs active hearing in conjunction with a bayesian-like means of integrating information over time can be robust to these ambiguities. More biologically precise models and electrophysiological recording in actively behaving animals would be necessary to establish the extent to which the avian or mammalian localization systems might employ such a strategy.

It is worth noting that our model resolves ambiguities associated with high-frequency sounds and the ITD cue, but it also resolves the front-back confusion at all frequencies (note that the spatial axis in the above figures span all 360 degrees of azimuth around the robot.) Although we did not include the interaural level-difference cue (ILD) nor ITDs carried by amplitude modulations in high-frequency sounds in our model, it is reasonable to predict that the Bayesian approach described here should likewise reduce ambiguities arising from front-back confusion regardless of the evidence cues used. In fact, a Bayesian updating approach is well-suited to fusing instantaneous bottom-up evidence from multiple cues. We therefore argue that future anatomical and physiological models of auditory localization should take an “active hearing” approach that explicitly includes head rotation and a Bayesian predictive processes.

The algorithm described above works by rotating the egocentric incoming evidence maps to align with the allocentric posterior probability map of the previous iteration. The parietal cortex of both rats [44] and monkeys [45] is thought to maintain both egocentric and allocentric representations of space and mediates the spatial transform required to move between the two representations. However, the low-level auditory system seems to use a strictly egocentric coordinate system. It should be noted that our model works equally well to resolve ambiguity if incoming evidence is recursively aligned to the egocentric frame. Computationally this is simply equivalent to rotating the allocentric prior probability map to align with the egocentric evidence map, and is conceptually more similar to a Kalman filter approach.

### A basis for spectral-spatial selective attention

Of particular interest is our finding that an ITD-sensitive system that employs a competitive process across spatial sources within frequency bands resolves complex acoustic scenes not only in space but in frequency as well. Thus, our model is capable of finding unique spectral-spatial locations dominated by a single sound source. Other computational approaches have been successful in finding the unique frequency bands occupied by individual talkers in a mixture. These approaches have been mainly confined to monaural mixtures [46–49] but binaural cues to separate sound sources have also been exploited successfully [34,50]. Lyon [51] used a dynamic spectral mask based on energy over very short intervals in binaural cross-correlation. They were able to achieve a rudimentary spectral separation of small samples but were limited by computational power. Roman et al [34] and Roman & Wang [52] have developed algorithms to find spectral masks that parse the spectrotemporal scene into discrete sources. Both systems make use of binaural cues to arrive at this spectral mask: Roman et al. [34] construct an estimate for an ideal binary mask by using a supervised learning approach. The system learns to use both ITD and IID cues to assess the degree of interference within a particular frequency band at a particular time. Time-frequency regions predicted to be dominated by the target are used to resynthesize the estimated target signal. Roman & Wang [52] developed a multi-stage algorithm to track moving sources using binaural input. This algorithm employed a binaural cross-correlation approach to identify spectral regions that reliably separated sources, followed by an HMM to identify likely transitions between different configurations of source location and interference. Similarly a system developed by Dietz et al. [33] uses ITD and ILD cues as well as a measure of instantaneous interaural envelope phase coherence to create a time-frequency mask that could localize up to five sources in the front field. Notably their approach also incorporated particle filtering to track sound source motion, but not head rotation. Our approach differs from these previous systems in that it simultaneously localizes and resolves the components of complex acoustic scenes in both the front and back field, while reducing the interference associated with ambiguity at high frequencies. However, an important limitation of our approach relative to the prior work is that we have not implemented a process by which “unreliable” time/frequency regions are selected against and removed from the final estimate of sound source locations. Thus our system can mislocalize, at least transiently, when two sound sources “collide” within a frequency band. In fact, our system will form a single stereo image when two sources are highly coherent in frequency and time. However, over time, the winner-take-all nature of the system tends to allow a single source to dominate in at least most frequency bands. A further difference between our system and prior work (e.g. [33,52]) is that our system does not actively track moving sources. In fact by aligning egocentric evidence to the allocentric reference frame, our system deliberately “blurs” any source that isn’t allocentrically stationary. By contrast, had we aligned prior evidence with the current egocentric reference frame, we would obtain a system that tracks sound sources moving with the head which, in real-world applications, could include noise from internal fans or motor components. Likewise, one might use both head rotation and a model of sound source motion to lock any number of “maps” to any number of targets. Conceptually this would be analogous the “update” step of a Kalman filter approach for target tracking.

## Conclusion

The results presented here expand our intuition about how the ITD cue might be used by biological systems to analyze the acoustic world. That understanding will be of subsequent interest with respect to computational auditory scene analysis (CASA), particularly for mobile or autonomous systems with movable microphone arrays. A common goal of CASA is to imitate the human ability to select target sound sources from a complex environment. Because the system described here resolves complex signals, such as speech or music, in both spatial and spectral domains, it may be possible to use the resulting probabilistic map as a mask to select only those frequency x spatial beams that are dominated by a target signal. We suggest that banks of frequency-tuned ITD units in both avian and mammalian auditory systems might be used for more than sound source localization; these systems might perform the computational "front-end" for an auditory selective attention system.

## Supporting information

S1 Dataset Dataset containing spectral-spatial map data from all experiments described. (ZIP)
